# The Imperial Yeomanry Hospital

**Published:** 1900-07-14

**Authors:** Alfred D. Fripp

**Affiliations:** Senior Surgeon I.Y.H.


### The Institutional Workshop.

### THE IMPERIAL YEOMANRY HOSPITAL.

By Alfred D. Fripp, M.S., F.R.C.S., Senior Surgeon I.Y.H.

(*Continued from page* 210.)

Thanks to the kindness of friends at home, there is a most excellent library and supply of periodicals. These are in great demand. The books are kept in cases in the recreation tent, and Mr. Atkins, one of the surgeons' dressers, performs the duties of librarian. Similarly, Mr. William Turner, one of the surgeons, presides over the distribution of all the indoor games throughout the hospital, and organises all the outdoor sports, including cricket, football, lawn tennis, golf, &c. All the requisites for these games have to be procured in Cape Town, and they are very expensive. The ground also does not lend itself at all favourably to the necessary preparation of level areas, so that we had great difficulties to overcome.

No. 14 represents a group of eight white "tortoise" tents, larger than the last, for they accommodate ten permanent bedsteads;

No. 15, the patients' lavatory and bath hut, which has a cement floor, sloping to a sink in the corner, and a good water supply;

No. 16, the hut in which all hospital food is cooked, and in which the cooks have stores and offices.

No. 17 is placed between the two acute dysentery wards, for which there is a special latrine, not for the use of patients, but for disinfection of stools, as will be described later on. On the plan a line is seen drawn round this dysentery department, and it shows that four of the "tortoise" tents mentioned under No. 14 are included. These four are used for the convalescent dysentery patients.

Above the dysentery department is shown another hut, and above that again the small hut used by Mr. Newland-Pedley as his store-room, work-room, and living-room, though the actual operative dental work is carried on in the second or spare operating theatre, to be described later.

Below the dysentery department are seen the huts that house No. 20, the pack store; and No. 21, the linen store.

No. 22 is placed between two huts.

No. 23 between the stewards or hospital store hut and the general store hut, in an annexe to which is housed the soda-water making machine;

No. 24 indicates the sergeants' mess tent;

No. 25, a group of five convalescent tents;

No. 26, the small tents, each of which house a convalescent officer;

No. 27, the officers' mess, into which opens No. 28, the officers' recreation-room, and close by which are:—

No. 29, the secretary's office;

No. 30, a small latrine;

No. 31, the orderly's room;

No. 32, the store and kitchen for the officers' mess; and

No. 33, the dairy, from which the milk for the entire camp is dispensed after sterilisation by several Aymard sterilisers. Miss Brereton, in addition to her work in the Operative Surgical Department, presides over the work of the dairy.

Half-way to the station is seen No. 34, the stable and coachhouse. A couple of Cape carts, each drawn by two horses, a cart which carries the linen backwards and forwards to the laundry, and the riding horses of the various officers, both medical and combatant, are here housed.

This leaves for description only the enteric department and the cross-shaped surgical building. The cross-shaped building labelled No. 35 in the plan shows the operating theatres, the surgery and dispensary, the *x* ray department, and the surgical store. The latter forms the head of the cross, and, with the two operating theatres, is in the charge of Miss Brereton, till lately the sister-in-charge of the pay wards at Guy's. In her surgical store are accumulated all the supplies of instruments, dressings, antiseptics, anæsthetics, splints, trusses, and other medical and surgical paraphernalia. It is separated by a roofed-in open-air lobby from the operating theatres, one of which is devoted, except in times of stress, to the dental surgeon. The two theatres form the cross-piece of the cross. The theatre used for surgical operations is most admirably equipped and furnished, Messrs. Down Brothers, who executed the order for all the instruments, having presented a complete set of aseptic metal furniture, including washhand stands, stools, dressing tables, and a splendid raise-and-lower table. The wood lining of the theatre has been painted white. There are two large slabs of corrugated glass which give an excellent top-light. The wooden floor is covered with linoleum; the doors are 4 ft. wide, giving easy access for the bearers from the open air or from the *x*-ray department. The long limb of the cross is also separated from the operating theatre by a roofed-in air space, and is occupied, as to the end nearest the operating theatre, by the *x*-ray department (which is singularly well equipped, and is doing most excellent work under Mr. Hall Edwards), and as to the foot of the cross by the surgery and dispensary. The photographic dark room is placed in the farthermost corner of the dispensary, so that the large store of plates shall not be spoiled by the proximity of the *x*-ray apparatus.

No. 36 is the enteric department. It consists of four huts, accommodating 22 to 26 patients each. In the centre of the department is a corrugated iron hut, half of which is used as a disinfecting latrine for the infected excreta, and the other half for the storing of used linen and bedding, including mattresses, until the regular hours which are appointed for the transference of these infected articles to the Washington-Lyons disinfector. All the bed-pans and urine utensils used in the department, and also all the water with which the patients have been washed, are carried by the orderlies to this enteric latrine, and there emptied into large pails containing Izal and disposed on the concrete floor, while on the zinc shelf there are sets of zinc baths, labelled A and B, for the thorough disinfection of all the utensils. In the baths labelled A the utensils are swabbed out with Izal solution, and then they are put to soak in a similar solution in the baths labelled B until required again in the wards. An intelligent native is perpetually on duty at the typhoid latrine swabbing out the walls, and floor, and iron shelves, which are all sloped especially to facilitate this process, while two other natives are continually on duty carrying the pails of the disinfected excreta away by the route indicated on the plan to a special dumping-ground in a natural hollow far removed from the camp. The pails brought back from the dumping-ground are fished out from a large tank of carbolic, and the dirty ones are put in their place, to undergo a similar soaking before they go back to the latrine.

The staff of attendants on duty in the typhoid department is entirely separate from the rest of the sisters and orderlies. Thus one of the senior sisters is in charge of the nursing staff, which consists of a day and a night sister for each hut, and a day sister for every two tents, and a night sister for every three tents. Similarly, a special ward master is in charge of the orderlies, of whom there are two on duty with each day sister, and one on duty with each night sister. The day staff and the night staff are each on duty for twelve hours in this department, their occasional holidays being arranged for by there being always one extra sister and two extra orderlies in addition to those already mentioned. By this means not only is efficiency conduced to among the attendants of the department, but also uniformity in the methods adopted for carrying out the rules, which have been printed and circulated to every attendant, and which are strictly enforced. There is a special hut between the two left-hand wards for the use of all sisters engaged in the department, and here they have their tea, &c., for taking of any food by the attendants, or even its preparation in the wards, is, of course, strictly forbidden. There is also a special lavatory for the enteric attendants, so that they may be able to disinfect their hands as frequently as they like.

As a postscript to this short account of the plan of our grounds, those interested in our hospital will be glad to hear that the longer we stop here the more pleased we are with the site. We are not free, of course, from many of the difficulties that beset all the military hospitals out here in common. For instance, we have to struggle with a very heavy visitation of enteric fever, which, by demanding the services of so many of our male and female staff, throws a heavy strain upon our resources, for we have only 100 orderlies to the 160 of a general military hospital. Also, our sisters often find it heart-breaking work to sustain the unequal battle against the never-ending invasion of vermin brought down by each train load from the front. We are harassed, too, by the fact that the supply of milk is precarious, and that eggs are extremely scarce. Fruit and fresh vegetables, except potatos and onions, are absolutely unobtainable. On the other hand, we have many things to be thankful for. Our water supply is unimpeachable. The climate is most healthy, sunny and warm in the day, and not very cold at night yet. The distant scenery is distinctly attractive. Our specialists in ophthalmic and dental surgery and $x$-ray work have proved a godsend. The same may be said with the utmost truth of our larger than usual body of nursing sisters. Nor must our masseur be forgotten. His assistance has been most efficacious in many cases of fractures and stiff joints and withered muscles. Again, it is a tremendous pull that we are able to do all our own washing, and that we have a really efficient steriliser, which can tackle, even though we are isolated in the Karroo, the otherwise unsurmountable difficulty of how to disinfect on a large scale.

Last, but by no means least, we owe much to our position of "splendid isolation." For nearly 30 miles on each side of us there are no signs of civilisation except a few ostrich and grazing farms. There are no troops, except the necessary guard of 50, to soil the ground, the water, or the air. For this reason we suffer less than most places from dust. We enjoy an immunity from outside interference, which will be readily appreciated by all those who are in a position to realise what an amount of hard work has to be got out of the staff, and how easily the harmonious working of a large volunteer staff, such as ours is, could be upset, or at least hampered, by the existence in our neighbourhood of any factors capable of still further complicating the many interests which have to be considered in the running of such a hospital.

Not a few ladies devoted to good works, but possessing no technical training, have come out to South Africa on purpose to "help in any capacity" in the military hospitals, but the explicit instructions we have received from the committee in London, for whom we act, no less than the fact that there is not a room which they could hire for their accommodation within 29 miles of us, has saved us from the unpleasant duty which might otherwise have devolved upon us of telling these willing, but unwanted, souls that, after a considerable experience of the value of their kindly-meant offers, and after the fullest consideration, we would very much rather have their room than their company.

P.S.—Of course, we should not have put enteric patients into the huts close to the operating theatre if we had not been flooded with them before we had any other accommodation available. But we have not had cause to regret this proximity. It is not always easy to forecast events accurately out here.

